# Perception of Beninese on intimate partner violence: evidence from 2011-2012 Benin demographic health survey

**DOI:** 10.1186/s12905-018-0633-x

**Published:** 2018-08-16

**Authors:** Alphonse Kpozehouen, Noël Moussiliou Paraïso, Yolaine Glèlè Ahanhanzo, Elvyre Klikpo, Charles Sossa Jérôme, Laurent T. Ouédraogo, Roger Salamon

**Affiliations:** 1 Division of Epidemiology and Biostatistics, Regional Institute of Public Health, Ouidah, Benin; 2 Division of Health Promotion, Regional Institute of Public Health, Ouidah, Benin; 3 Faculty of Health Sciences, Cotonou, Benin; 4 Division of Health Promotion, Regional Institute of Public Health, Ouidah, Benin; 5 Institute of Public health, Epidemiology and development, Bordeaux, Bordeaux, France; 6 Cotonou, Benin

**Keywords:** Intimate partner violence, Social perception, Benin

## Abstract

**Background:**

Violence against women remains an important issue of inequality in African societies, with several consequences to health, social and economic status. This study aims to identify the factors related to the perception of intimate partner violence in Benin.

**Methods:**

Data on intimate partner violence was collected by conducting live interviews, and from the Benin Demographic and Health Survey 2012. The dependent variable was acceptance of intimate partner violence. The independent variables were socio-demographic features such as age, level of education, matrimonial status, ethnicity, religion, place of residence and the index of economic well-being. Logistic regressions were performed and odds ratios (OR) with a confidence interval of 95% (CI_95%_) were estimated.

**Results:**

Among the 21,574 people who answered the questions relating to violence against women by an intimate partner, the prevalence of acceptance of intimate partner violence was 15.77%. Ethnicity, level of education, administrative department of residence, religion, and socio-economic quintile were factors associated with the respondents’ acceptance of violence against women by an intimate partner.

**Conclusion:**

Acceptance of intimate partner violence could be a major obstacle to the success of some health programs. There is a need to break the norms that support the vulnerability of women in Beninese society.

## Background

Intimate partner violence (IPV) is a human rights violation. It is a form of discrimination towards women that, both in law and in fact, conveys the persistence of inequalities between men and women. IPV is also a public health issue that could have consequences to women’s physical and mental health [1–3]. According to the World Health Organization (WHO), the global prelavence of IPV is 30%, and this prevalence is very high in countries in Sub-Saharan Africa and Southeast Asia [4–6].

IPV is known to be associated with both short- and long-term psychological and mental problems and health issues, including depression, anxiety, and tendencies towards addiction and suicidal thoughts [7–9]. Some authors have tried to explain the circumstances surrounding IPV [10]. In African societies, there is a pre-established order in which the woman must submit to her husband or spouse. However, we are now noticing that economic development among women, their education and their financial autonomy are making them more aware of gender inequalities. This awareness in turn leads to conflicts. Violence against women therefore seems to be one of the most brutal consequences of the economic, social, political and cultural inequalities that exist between men and women [11]. In Benin, despite the existence of a legislative [12] and regulatory framework (Individuals and Family Code) that protect the rights of vulnerable people such as women, IPV is still observed. The upsurge of this phenomenon is such that, according to a survey undertaken by the Ministry of Family and National Solidarity in 2009, 69% of Beninese women had suffered abuse at least once in their life [13]. Abuse against women in Benin takes multiple and varied forms: from sexist insults to psychophysiological abuses, through forced marriages (by abduction or exchange) or religious sequestration, etc. The causes of such abuse must be investigated within the society or culture of the perpetrators and the victims. Abuse against women is based on a society’s perceptions, not only of violence, but also of men and women. The purpose of the present study is to assess women and men’s perception of IPV by determining the prevalence and associated factors of this phenomenon in Benin.

## Methods

### Study description

Located in Western Africa, in the Gulf of Guinea, Benin has a surface area of 114,763 km^2^. Its population was estimated at 8,364,942 in 2008 based on the projections of the 2002 census [14]. Its main economic activities are agriculture, the craft industry, and informal trade. The organization of the healthcare system is based on the primary healthcare model with a central level, an intermediate level, and a peripheral level encompassing all healthcare programs.

### Data

Data on health and intimate partner violence in Benin were collected as part of a secondary Benin Demographic and Health Survey (DHS) which took place in 2011–2012 [15]. The Benin DHS contained datasets of adult men in the 15–64 age group and women in the 15–49 age group. The Benin DHS data were collected using interview methods compliant with international and national ethical guidelines. The Benin DHS was designed to provide socio-demographic and health indicators at urban, rural and regional levels. The Benin DHS samples were selected using a stratified two-stage cluster sampling design. Sampling of women and men was performed according to the list of enumeration areas developed from the 2002 Population Census sampling frame. The initial sampling stage involved the selection of 750 clusters, also known as Primary Sampling Units (PSUs) with a probability proportional to the size. The size, in this case, is the number of households in the cluster. Data were collected through face-to-face interviews with 16,599 women aged 15 to 49 years, and with 5180 men aged 15 to 64 years.

### Ethical considerations

Permission to use the above-mentioned data in our study was obtained from ORC Macro Inc., and approval was obtained from the National Ethics Committee in the Benin Ministry of Health, and the Ethics Committee of the Opinion Research Corporation Macro International, Inc. (ORC Macro Inc., Calverton, MD, USA).

### Instrument

#### Dependent variable

Participants were asked whether a husband/partner is justified in abusing his wife/partner under the following series of circumstances: i) “the woman burned the food”; ii) “the woman argued with the man”; iii) “the woman went out of the home without prior permission from her partner”; iv) “the woman neglected the children”; and v) “the woman refused to have sex with the man”. The response format to these questions was “yes” or “no” [16].

A binary outcome variable was created for acceptance of IPV based on yes and no; if the respondent did not agree with any of the circumstances mentioned or did not have any opinion on the issue, the answer was “No”, and if the respondent agreed with at least one of the circumstances mentioned above, the answer was “Yes”.

#### Independent variables

The independent variables considered were:

- The age of the interviewed persons, categorized into 3 groups: ≤24; 25–34 and ≥ 35.
- The level of education of both the men and the women, classified in 4 categories: i) No education; ii) Primary education; iii) Secondary education; iv) Higher education.
- The matrimonial status of the survey participants, categorized as: i) Never been in a relationship; ii) Married; iii) Living with partner; iv) Widowed; v) Divorced; vi) No longer living together.
- The religion practiced by the respondents, classified as: i) Voodoo/Traditional; ii) Islam; iii) Christian; iv) Other religion; v) No religion.
- The occupation of the survey participants, classified as: i) “Working”, if the person worked; ii) “Not working”, if they did not work.
- The living area of the respondents, categorized as: i) Rural; ii) Urban.
- The place of residence, i.e. in which of the 12 administrative departments of Benin the survey participants resided.
- The variable “who makes decisions regarding household expenditure”, categorized as: i) The woman; ii) The woman or the man; iii) The man

In the absence of reliable data on income and expenditure in developing countries, in the Demographic and Health Surveys, the poverty index is used. It is a composite index or indicator of the socio-economic status of households that assigns weightings or factor scores generated by the principal component analysis to information collected on household assets. Thus, each respondent was ranked according to the household asset score and was assigned to wealth quintiles as follows: the poorest, the second poorest, the average, the second wealthiest and the wealthiest [17]. Despite its limitations, the wealth index is usually accepted as a fairly good measure of the economic situation and is used as a proxy indicator for income [18].

### Statistical analysis

For the descriptive analysis, we compared the independent qualitative variables with the dependent variable “acceptance of IPV” using Pearson’s χ^2^ test.

The averages were compared with the Student t-test or with the analysis of variance.

The factors linked to the acceptance of IPV were selected at 20% in the univariate analysis, and the interaction terms identified in the stratified analysis were used in a multivariate logistic regression model taking into account the cluster effect to identify the potential risk factors of the acceptance of IPV.

The associations between the acceptance of IPV and the other variables were assessed by odds ratios (OR) with a confidence interval of 95% (CI_95%_).

For all the analyses, the study took into account the weight of each PSU (Primary Sampling Unit) [19].

## Results

### Main descriptive statistics

A total of 21,574 people answered the questions relating to intimate partner violence (IPV), with 5145 men and 16,429 women. The rate of non-response was 0.95%. The mean age of the population was 29.84 ± 12.73 years with a minimum of 15 and a maximum age of 64 respectively. The average age for men was 32.86 years and the average age for women was 28.91 years. The prevalence of the acceptance of IPV was 15.77% [14.78–16.75]. The prevalence of the acceptance of IPV was 16.2% among women and 14.4% among men; the ethnic group in which acceptance of IPV was the highest was the Peulh (30.4%), followed by the Bariba (20.6%). Acceptance of IPV among the Yoruba and the respondents with the highest level of education was less at 12.1% and 4.1% respectively. IPV acceptance is around 15–16% across all age groups. The married or divorced respondents were the groups with the highest prevalence of acceptance of IPV at around 18% (17.7% and 18.1% respectively). In rural areas, 17% of the respondents accepted IPV; the departments of Borgou and Plateau were the ones with the extreme values of prevalence of IPV acceptance at 25.8% and 9.8%. As concerns religion, the respondents who followed traditional religions and those who followed Islam had the highest prevalence: 19.4% and 18.5%. (Table 1).

**Table 1:** Association Between Respondent’s Socio-Demographic Characteristics and Perception of Intimate Partner Violence, BDHS, 2012

	Number (n)	Intimate partner violence (%)	*P*-value
Gender			0.102
Man	5145	14.4	
Woman	16,429	16.2	
Ethnicity			0.0000
Adja	3302	15.6	
Bariba	1984	20.6	
Dendi	787	16.5	
Fon	9575	15.6	
Yoa	817	18.8	
Betamari	1437	18.2	
Peulh	808	30.4	
Yoruba	2,53	12.1	
Other Beninese	87	21.6	
Other nationality	247	19.2	
Level of education			0.0000
No education	12,317	18.6	
Primary	3938	14.5	
Secondary	4785	11.7	
Higher	534	4.1	
Age Group (year)			0.2518
15–24	7276	15.1	
25–34	7051	16.4	
35 years et +	7247	15.9	
Matrimonial Status			0.0000
Never in relation	5544	13.9	
Married	11,692	17.7	
Living with partner	3307	13.7	
Widowed	337	14.5	
Divorced	159	18.1	
No longer living together	535	14.1	
Profession			0.2804
No working	7306	14.8	
Working	13,438	15.6	
Area			0.0033
Rural	12,464	17.0	
Urban	9,11	14.1	
Department			0.0000
Alibori	1328	11.8	
Atacora	1825	14.6	
Atlantique	2482	19.7	
Borgou	1757	25.8	
Collines	1,68	18.0	
Couffo	1544	16.5	
Donga	1231	16.3	
Littoral	2485	14.8	
Mono	1349	16.5	
Ouémé	2405	14.4	
Plateau	1354	9.8	
Zou	2134	16.3	
Religion			0.0000
Voodoo/Traditional	3083	19.4	
Islam	5176	18.5	
Christian	11,864	14.3	
Other Religion	418	13.1	
No Religion	1033	14.7	
Quintile			0.0000
Poorest	4146	17.7	
Poorer	4411	17.1	
Middle	4469	17.5	
Richer	4,69	14.3	
Richest	3858	12.7	
Who makes spending decisions?			0.0045
The woman	1466	20.61	
The woman or the man	2839	16.04	
The man	9983	15.44	

Note. BDHS = Benin Demographic Health Survey

### Univariate and multivariate analysis (Table 2)

Concerning the socio demographic features, the prevalence of acceptance of IPV was higher among women than among men, but this association was not significant (OR = 1.14; CI_95%_ = [0.97; 1.35]). The risk of IPV acceptance among women from the Peulh and Bariba groups was, respectively, 2 and 1.41 times higher than for women of Adja ethnicity (OR = 2.36; CI_95%_ = [1.67; 3,34] and OR = 1.41; CI_95%_ = [1.12; 1.77] respectively). On the other hand, women living in Yoruba were at low risk of being abused by their intimate partner (OR = 0.74; CI_95%_ = [0.55; 0.99]). Acceptance of IPV was very high among the uneducated respondents compared to those with a higher level of education. The respondents who received no education were at higher risk of IPV acceptance than those with a higher level of education (Table 2). Age was observed to be independent of the acceptance of IPV. The risk of acceptance of IPV was observed to be 1.32 times higher among the married respondents than the ones who had never been in a relationship (OR = 1.32; CI_95%_ = [1.18; 1.49]). In rural areas, more of the respondents accepted IPV compared to the respondents in urban areas (*p*-value = 0.0033). The respondents thought that women in the following areas could be subjected to IPV: the administrative departments of Borgou (25.8%), Atlantique (19.7%), Collines (18.0%), Couffo and Mono (16.5%). Within each of these five departments, women were indeed at a higher risk of being the victims of IPV than those in the department of Alibori (Table 2). As far as religion is concerned, compared to the Christian religion, those who follow Voodoo/Traditional practices and Islam were indeed more at risk of accepting IPV (OR = 1.43; CI_95%_ = [1.21; 1.70] and OR = 1.35; CI_95%_ = [1.17; 1.56] respectively). In the poorest class, the respondents thought that men were justified in abusing their wives/partners. This justification of IPV increased from the poorer to the wealthier respondents (Table 2). In the households where women made decisions regarding household expenses, acceptance of IPV was higher (OR = 1.42; CI_95%_ = [1.14; 1.77]) than in the households where men made such decisions. The results of the multivariate analysis (Table 2) show that more of the respondents from the Peulh and Betamari ethnic groups thought that IPV was justified compared to those from the Adja ethnic group, independently of the other variables of the model. The respondents with a low level of education showed wide acceptance of IPV. The risk was highest among the respondents with no education (OR = 5.36; CI_95%_ = [3.62; 7.95]). However, among the respondents with a primary level of education, the risk was lower (OR = 4.07; CI_95%_ = [2.72; 6.10]). The respondents with a secondary level of education had a higher risk (OR = 3.21; CI_95%_ = [2.15; 4.78]) compared to those with a high level of education. According to the multivariate analysis shown in Table 2, the participants who practiced Voodoo or a Traditional religion showed greater acceptance of IPV compared to the Christian respondents (OR = 1.27; CI_95%_ = [1.07; 1.51]). The respondents who practiced Islam also approved of IPV (OR = 1.25; CI_95%_ = [1.03; 1.52]). Compared to the households in the highest socio-economic quintile (meaning the wealthiest), the poorest respondents (quintile 1) and those belonging to the middle class (quintile 3) feasibly accepted IPV (OR = 1.25; CI_95%_ = [1.01; 1.54] and (OR = 1.31; CI_95%_ = [1.09; 1.56] respectively).

**Table 2:** Multivariate Logistic Regression Analysis of Predictors of Intimate Partner Violence: BDHS, 2012

	Crude Odds Ratio	CI 95%		*P*-value	Adjusted Odds Ratio	CI 95%		*P*-value
Gender								
Man	1							
Woman	1.14	0.97	1.35	0.102				
Ethnicity								
Adja	1				1			
Bariba	1.41	1.12	1.77	0.003	1.03	0.70	1.52	0.858
Dendi	1.07	0.77	1.47	0.676	1.28	0.85	1.95	0.231
Fon	1.00	0.83	1.20	0.984	1.21	0.93	1.58	0.152
Yoa	1.25	0.88	1.79	0.203	1.53	0.98	2.40	0.059
Betamari	1.20	0.90	1.61	0.199	1.57	1.06	2.31	0.022
Peulh	2.36	1.67	3.34	0.000	1.67	1.02	2.76	0.041
Yoruba	0.743	0.55	0.99	0.047	1.05	0.76	1.45	0.757
Other Beninese	1.49	058	3.83	0.404	1.52	0.58	3.97	0.384
Other nationality	1.28	0.85	1.93	0.222	1.42	0.92	2.18	0.105
Level of education								
No education	5.36	3.56	8.05	0.000	5.36	3.62	7.95	0.000
Primary	3.97	2.62	6.02	0.000	4.07	2.72	6.10	0.000
Secondary	3.10	2.05	4.69	0.000	3.21	2.15	4.78	0.000
Higher	1				1			
Age Group (year)								
15–24	1							
25–34	1.09	0.97	1.23	0.120				
35 years et +	1.05	0.95	1.17	0.305				
Matrimonial Status								
Never in relation	1							
Married	1.32	1.18	1.49	0.000				
Living with partner	0.98	0.80	1.19	0.876				
Widowed	1.05	0.72	1.51	0.786				
Divorced	1.36	0.85	2.18	0.187				
No longer living together	1.01	0.74	1.38	0.906				
Profession								
No working	1							
Working	1.06	0.95	1.19	0.280				
Area								
Rural	1.24	1.07	1.44	0.003				
Urban	1							
Department								
Alibori	1				1			
Atacora	1.28	0.92	1.78	0.131	1.33	0.88	2.01	0.166
Atlantique	1.83	1.34	2.50	0.000	2.70	1.80	4.04	0.000
Borgou	2.60	1.96	3.45	0.000	3.20	2.32	4.40	0.000
Collines	1.65	1.23	2.20	0.001	2.48	1.71	3.62	0.000
Couffo	1.48	1.08	2.03	0.014	2.23	1.40	3.55	0.001
Donga	1.46	1.02	2.10	0.039	1.46	0.96	2.21	0.070
Littoral	1.30	0.98	1.74	0.068	2.78	1.84	4.18	0.000
Mono	1.47	1.02	2.13	0.036	2.46	1.54	3.92	0.000
Ouémé	1.26	0.93	1.71	0.126	1.92	1.29	2.83	0.001
Plateau	0.81	0.51	1.29	0.384	1.14	0.68	1.91	0.602
Zou	1.45	1.06	1.99	0.018	2.01	1.32	3.06	0.001
Religion								
Voodoo/Traditional	1.43	1.21	1.70	0.000	1.27	1.07	1.51	0.005
Islam	1.35	1.17	1.56	0.000	1.25	1.03	1.52	0.022
Christian	1				1			
Other Religion	0.90	0.64	1.27	0.561	0.82	0.58	1.17	0.289
No Religion	1.03	0.83	1.28	0.768	0.88	0.70	1.09	0.259
Quintile								
Poorest	1.48	1.21	1.81	0.000	1.25	1.01	1.54	0.034
Poorer	1.42	1.18	1.71	0.000	1.21	0.99	1.47	0.056
Middle	1.46	1.23	1.74	0.000	1.31	1.09	1.56	0.003
Richer	1.15	0.98	1.34	0.072	1.09	0.93	1.28	0.244
Richest	1				1			
Who makes spending decisions?								
The woman	1.42	1.14	1.77	0.002				
The woman or the man	1.04	0.88	1.23	0.594				
The man	1							

Note. CI = Confidence Interval, BDHS = Benin Demographic Health Survey

**Fig. 1:**
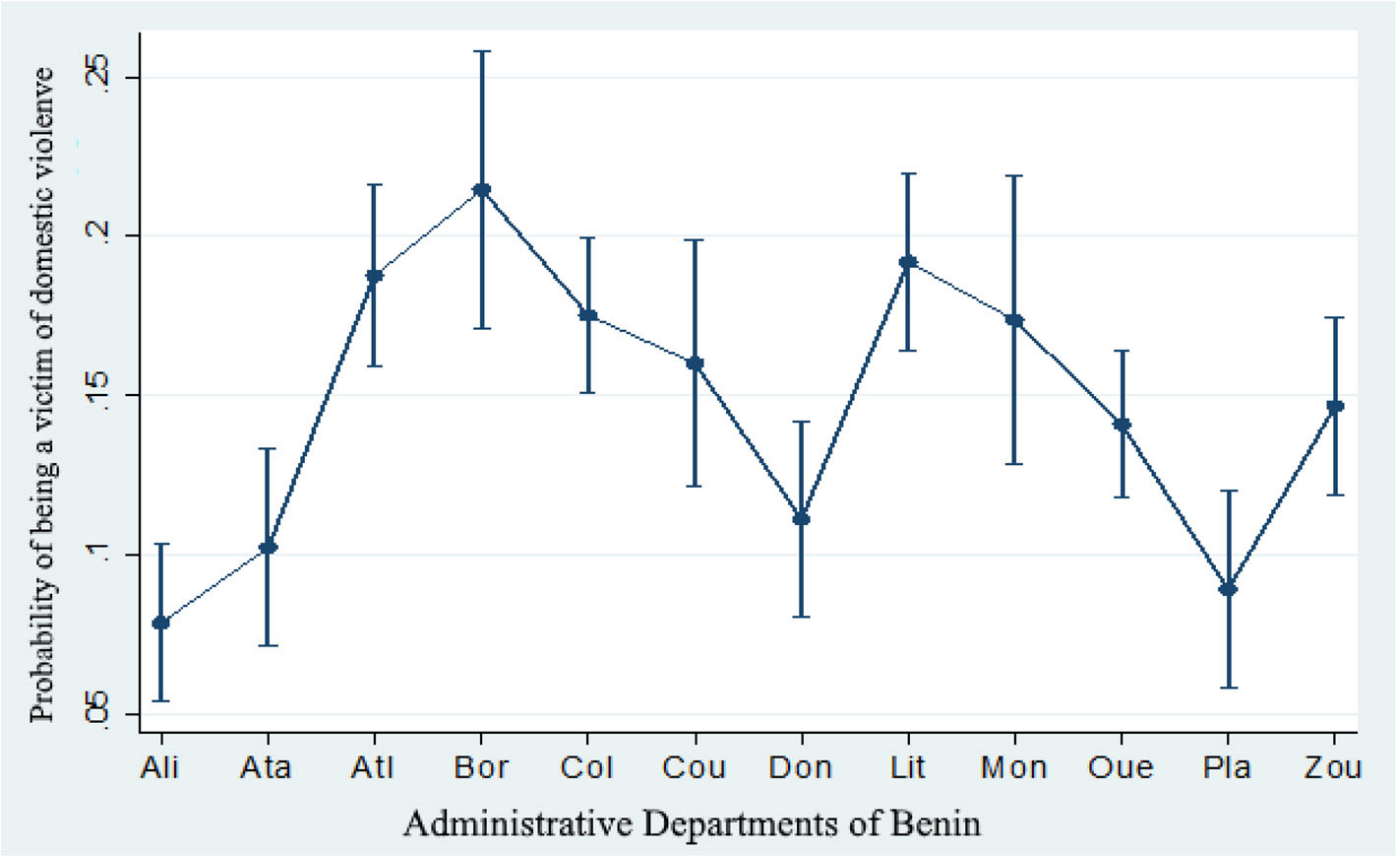
Probability of accepting “Intimate partner violence” in Benin: BDHS, 2012. **Ali** = Alibori **Ata** = Atacora **Atl** = Atlantique **Bor** = Borgou **Col** = Collines **Cou** = Couffo **Don** = Donga **Lit** = Littoral **Mon** = Mono **Oue** = Ouémé **Pla** = Plateau **Zou** = Zou

By adjusting the dependent IPV variable for the independent variables, the variables retained in the final model were ethnicity, level of education, administrative department, religion and socio-economic quintile. According to Fig. 1, the administrative departments with the highest probability of IPV were Borgou (21.4%), followed by Littoral (19.2%) and Atlantique (18.8%). The probability was the lowest in the departments of Alibori (7.9%), Plateau (8.9%) and Atacora (10.2%).

## Discussion

This study presents the first national estimates of violence against an intimate partner in Benin, using data from a population-based probability sample.

According to the results of this study, one participant out of six (15.77%) considered that it was justified for a woman to suffer abuse from her husband/partner for at least one of the following reasons: she burned the food, argued with her partner, went out of the house without notifying her partner, neglected the children, or refused to have sex with her partner. Many socio-demographic factors were related to the probability of acceptance of IPV. Ethnicity, level of education, administrative department of residence, religion, and socio-economic quintile were independently associated with the respondents’ acceptance of IPV.

In the univariate analysis, the respondents of Yoruba ethnicity were less accepting of IPV: their acceptance was 12.1%. This result corroborated the study of Antai et al. and Oyediran et al. in Nigeria where Yoruba women have a certain degree of autonomy [20, 21]. In Benin, within the Yoruba ethnic group, women are often financially self-sufficient, which gives them a certain respect for themselves and explains the low rate of IPV.

Most of the respondents who approved IPV belonged to the Peulh and Betamari ethnicities. Their positive perception of IPV was due to the existence of certain sociological factors within their societies that increase women’s vulnerability to violence. The following are some of those factors: the dowry (which is still expensive within the two ethnic groups); the integrity of tradition which forces women to be submissive or makes them accept abusive activities from their husband/partner; religion and beliefs (the Peulh are mostly Muslims, whereas the Betamari practice traditional religions); and violence, which is mostly a cultural inheritance of the Peulh [13]. The less educated the respondent was, the more likely they were to agree with abuses against women. Similar results had been found in Ghana and Malawi [22, 23]. A high level of education would therefore reduce acceptance of IPV. The importance and necessity of putting emphasis on education is clearly evident. As long as respondents do not have a high level of education, they will not be able to assess the consequences of abuses against women [24]. In accordance with studies carried out in Uganda [25], Kenya [26] and in Ghana [27], the results of this study also show that residing in a rural area increases the risk of accepting IPV. This could be explained by the traditional view of gender roles. In rural areas, where traditional values are dominant, women are expected to take care of the household and children, and to show obedience and respect to their husbands/partners. In these traditional societies, it is considered that men who beat their wives/partners are entitled to do so. The respondents who practiced Islam or traditional religions showed greater acceptance of IPV compared to those who practiced the Christian religion. Our results match those of other authors from Ghana and from Arab and Islamic countries [27, 28]. This acceptance of IPV could be explained by the socio-cultural constraints observed in patriarchal systems, and in societies based on customary and religious beliefs and practices. Religious and traditional leaders should therefore be called upon to assist in the fight against IPV; they can teach the respondents about gender tolerance. Respondents from poorer households ran a higher risk of accepting IPV. Similar results have also been found in Ghana [23, 24]. The prevalence of IPV was particularly high in the administrative departments of Borgou, Atlantique and Littoral. The Beninese authorities have yet to investigate this difference between the departments. Discovering its underlying causes will allow them to take targeted action to protect women in those departments.

### Limitations and strengths of the study

Though this study was strong enough, one of its limitations is the use of an indirect index of economic well-being. In low- and middle-income countries it is difficult to obtain reliable income or expenditure data. An asset-based index is generally considered a good indicator of household wealth.

Our study focused on understanding the role of individual variables as determinants of the attitude towards IPV. However, in the design of this study, we did not include an assessment of the effect of interactions between those variables and other social factors: for example, social variables such as the level of education or wealth of each region. Future studies using a multi-level design will be necessary in order to take those considerations into account.

Other matters such as the causes of domestic violence due to non-domestic factors (such as the woman’s financial status or the husband/partner’s intoxication) were not included in the measurement of IPV. Beside the validity of the data collection tools used, the potential limitations of the face-to-face interview method must be acknowledged [29]. For example, compared with self-administered questionnaires, respondents may tend not to fully disclose their attitude towards IPV in the presence of interviewers. Nevertheless, ethical measures such as guarantees of anonymity and the administration of interviews by trained staff have made it possible to improve these reports [29].

Despite the previously mentioned limitations, the strengths of this study are significant. The Demographic and Health Survey (DHS) is a major study of the Beninese population at a national scale. Furthermore, the DHS data is largely recognized as being of high quality because they are based on a rigorous and precise sampling methodology with a high response rate. During the data collection of the DHS, strict ethical rules were also employed for IPV.

## Conclusion

This study has enabled us to identify the individual factors that could explain acceptance of IPV. There is a need for proactive efforts to break the norms that support the vulnerability of women in Beninese society. Direct concerted efforts from both the Beninese government and from non-governmental organizations are necessary to raise awareness on IPV and, if possible, to challenge certain social norms (such as the superiority of men over women). The Beninese authorities can reduce the vulnerability of women by promoting the education of both men and women. The assistance of religious authorities could also be requested.

This study has provided information on potential individual factors related to IPV in Benin. However, knowledge about the contextual factors linked to IPV is still limited.

## References

[1] Ellsberg M, Jansen HA, Heise L, Watts CH, Garcia-Moreno C (2008). Intimate partner violence and women's physical and mental health in the WHO multi-country study on women's health and domestic violence: an observational study. Lancet (London, England).

[2] Garcia-Moreno C, Watts C (2011). Violence against women: an urgent public health priority. Bull World Health Organ.

[3] Watts C, Zimmerman C (2002). Violence against women: global scope and magnitude. Lancet (London, England).

[4] Organisation mondiale de la Santé/London School of Hygiene and Tropical Medicine (2010). Prévenir la violence exercée par des partenaires intimes et la violence sexuelle contre les femmes: intervenir et produire des données.

[5] Peltzer K, Pengpid S (2014). Female genital mutilation and intimate partner violence in the Ivory Coast. BMC Womens Health.

[6] WHO (2013). Intimate partner and sexual violence against women. In.

[7] Rahman M, Nakamura K, Seino K, Kizuki M (2012). Intimate partner violence and use of reproductive health services among married women: evidence from a national Bangladeshi sample. BMC Public Health.

[8] Rodriguez MA, Heilemann MV, Fielder E, Ang A, Nevarez F, Mangione CM (2008). Intimate partner violence, depression, and PTSD among pregnant Latina women. Ann Fam Med.

[9] Zlotnick C, Johnson DM, Kohn R (2006). Intimate partner violence and long-term psychosocial functioning in a national sample of American women. J Interpers Violence.

[10] Rani M, Bonu S, Diop-Sidibe N (2004). An empirical investigation of attitudes towards wife-beating among men and women in seven sub-Saharan African countries. Afr J Reprod Health.

[11] Beridogo B (2002). Etudes sur les violences faites aux femmes.

[12] de la République P. Loi N°2011–26 du 09 Janvier 2012 portant prévention et répression des violences faites aux femmes. Bénin; 2012.

[13] Ministère de la Famille et de la Solidarité Nationale (2009). Les violences faites aux femmes au Bénin. In.

[14] Institut national de la statistique et de l’analyse économique du Bénin (2002). Rapport du 3e recensement général de la population et de l’habitat au Bénin en 2002.

[15] Institut National de la Statistique et de l’Analyse Économique (INSAE) et ICF International (2013). Enquête Démographique et de Santé du Bénin 2011–2012.

[16] Uthman OA, Lawoko S, Moradi T (2010). Sex disparities in attitudes towards intimate partner violence against women in sub-Saharan Africa: a socio-ecological analysis. BMC Public Health.

[17] Rutstein S, Kiersten J. The DHS Wealth Index. DHS Comparative Reports No. 6. Calverton, Maryland; 2004.

[18] Vyas S, Kumaranayake L (2006). Constructing socio-economic status indices: how to use principal components analysis. Health Policy Plan.

[19] Bennett S, Woods T, Liyanage WM, Smith DL (1991). A simplified general method for cluster-sample surveys of health in developing countries. World Health Stat Q.

[20] Antai DE, Antai JB (2008). Attitudes of women toward intimate partner violence: a study of rural women in Nigeria. Rural Remote Health.

[21] Oyediran KA, Isiugo-Abanihe U (2005). Perceptions of Nigerian women on domestic violence: evidence from 2003 Nigeria demographic and health survey. Afr J Reprod Health.

[22] Conroy A (2014). Gender, power, and intimate partner violence: a study on couples from rural Malawi. J Interpers Violence.

[23] Doku DT, Asante KO (2015). Women's approval of domestic physical violence against wives: analysis of the Ghana demographic and health survey. BMC Womens Health.

[24] Mann J, Takyi B (2009). Autonomy, dependency or culture: examining the impact of resource and socio-cultural processes on attitudes towards intimate partner violence in Ghana. Africa J Fam Violence.

[25] Ogland E, Xu X, Bartkowski J, Ogland C (2014). Intimate partner violence against married women in Uganda. J Fam Violence.

[26] Odero M, Hatcher A, Bryant C, Onono M, Romito P, Bukusi E (2014). Responses to and resources for intimate partner violence: qualitative findings from women, men, and service providers in rural Kenya. J Interpers Violence.

[27] Colucci E, Hassan G (2014). Prevention of domestic violence against women and children in low-income and middle-income countries. Curr Opin Psychiatry.

[28] Douki S, Nace F, Belhad A, Bouasker A, Ghachem R (2003). Violence against women in Arab and Islamic countries. Arch Womens Ment Health.

[29] Lawoko S (2008). Predictors of attitudes toward intimate partner violence: a comparative study of men in Zambia and Kenya. J Interpers Violence.

